# Crystal orientation-dependent tensile mechanical behavior and deformation mechanisms of zinc-blende ZnSe nanowires

**DOI:** 10.1038/s41598-023-30601-3

**Published:** 2023-03-02

**Authors:** A. S. M. Jannatul Islam, Md. Sayed Hasan, Md. Sherajul Islam, Ashraful G. Bhuiyan, Catherine Stampfl, Jeongwon Park

**Affiliations:** 1 Department of Electrical and Electronic Engineering, Khulna University of Engineering and Technology, Khulna, 9203 Bangladesh; 2 School of Physics, The University of Sydney, Sydney, NSW 2006 Australia; 3 Department of Electrical and Biomedical Engineering, University of Nevada, Reno, NV 89557 USA; 4 School of Electrical Engineering and Computer Science, University of Ottawa, Ottawa, ON K1N 6N5 Canada

**Keywords:** Engineering, Materials science, Nanoscience and technology

## Abstract

Crystal deformation mechanisms and mechanical behaviors in semiconductor nanowires (NWs), in particular ZnSe NWs, exhibit a strong orientation dependence. However, very little is known about tensile deformation mechanisms for different crystal orientations. Here, the dependence of crystal orientations on mechanical properties and deformation mechanisms of zinc-blende ZnSe NWs are explored using molecular dynamics simulations. We find that the fracture strength of [111]-oriented ZnSe NWs shows a higher value than that of [110] and [100]-oriented ZnSe NWs. Square shape ZnSe NWs show greater value in terms of fracture strength and elastic modulus compared to a hexagonal shape at all considered diameters. With increasing temperature, the fracture stress and elastic modulus exhibit a sharp decrease. It is observed that the {111} planes are the deformation planes at lower temperatures for the [100] orientation; conversely, when the temperature is increased, the {100} plane is activated and contributes as the second principal cleavage plane. Most importantly, the [110]-directed ZnSe NWs show the highest strain rate sensitivity compared to the other orientations due to the formation of many different cleavage planes with increasing strain rates. The calculated radial distribution function and potential energy per atom further validates the obtained results. This study is very important for the future development of efficient and reliable ZnSe NWs-based nanodevices and nanomechanical systems.

## Introduction

It has recently been demonstrated experimentally^1,2^ and theoretically^3,4^ that the mechanical behavior in semiconductor nanowires (NWs) exhibits a strong orientation dependence. Significant anisotropic mechanical properties along different crystal orientations are observed in NW systems^5–7^. Other physical properties, including electrical and thermal conductivity, piezoelectric polarization, refraction index, surface reactivity, and bandgap can be precisely altered by controlling the crystal growth directions^2,8–10^. There are differences among the side surfaces in wurtzite/zinc-blende NWs that grow in different directions. These differences can significantly alter the deformation mechanism over perfect and partial dislocation slipping and deformation twinning^11–15^. Moreover, when crystal orientation is coupled with temperature and strain rate, different deformation mechanisms occur in NWs due to competition between global and local deformations, activation variation of different planes, and influence disparity of inter-planar distances^16–21^. Thus, a deeper understanding of the crystal orientation-dependent properties is essential for the production and functionality of NWs in their real-world applications.

On the other hand, the unique applications, microscopic physics, and fabrication of nanoscale devices, such as laser diodes, photodetectors, field effect transistors (FETs), and solar cells, make semiconductor NWs extremely promising in these research fields. In particular, zinc-blend ZnSe NWs^22^ have garnered substantial attention as next-generation nanoelectronic materials owing to their outstanding performance with mechanical flexibility, transmittance, conductivity, and inexpensive synthesis^23–26^. In addition, the direct bandgap (~ 2.7 eV), high absorption coefficient, suitable electronegativity, and unique nonlinear properties of zinc-blende ZnSe make it potentially an important material for blue lasing applications, optical waveguides, thermoelectric systems, light-emitting diodes, nanosensors, nanoactuators, nanoresonators, and magnetic information storage^27–37^. However, despite considerable effort to assess the electronic, thermal, and optical characteristics of zinc-blende ZnSe NWs, both in theory^23,24,26,38,39^ and in experiments^40–46^, to the best of our knowledge, there is no study on their mechanical properties. In particular, the tensile deformation mechanisms for different crystal orientations have not been reported in the literature. Furthermore, the mechanical strength variation under diverse external variables such as temperature and strain rate coupled with orientation is also unknown.

In this work, we have employed molecular dynamics (MD) simulations to expose the influence of crystal orientations such as [100], [110], and [111] at different temperatures (ranging from 100 to 600 K) and strain rates (varying from 1 × 10^8^ s^−1^ nm to 1 × 10^10^ s^−1^) on the tensile mechanical properties and deformation mechanisms of zinc-blende ZnSe NWs. To describe the atomistic interaction of zinc-blende ZnSe NWs systems, the classical Stillinger–Weber (SW) potential developed by Zhou et al.^47^ was utilized. In addition, we have calculated the radial distribution function and potential energy per atom to elucidate the orientation-dependent mechanical behavior and deformation mechanisms. This work clarifies the dependence of crystal orientation on the mechanical properties of ZnSe NWs and brings practical applications of ZnSe-based nanodevices one step further.

## Computational details

**Figure 1:**
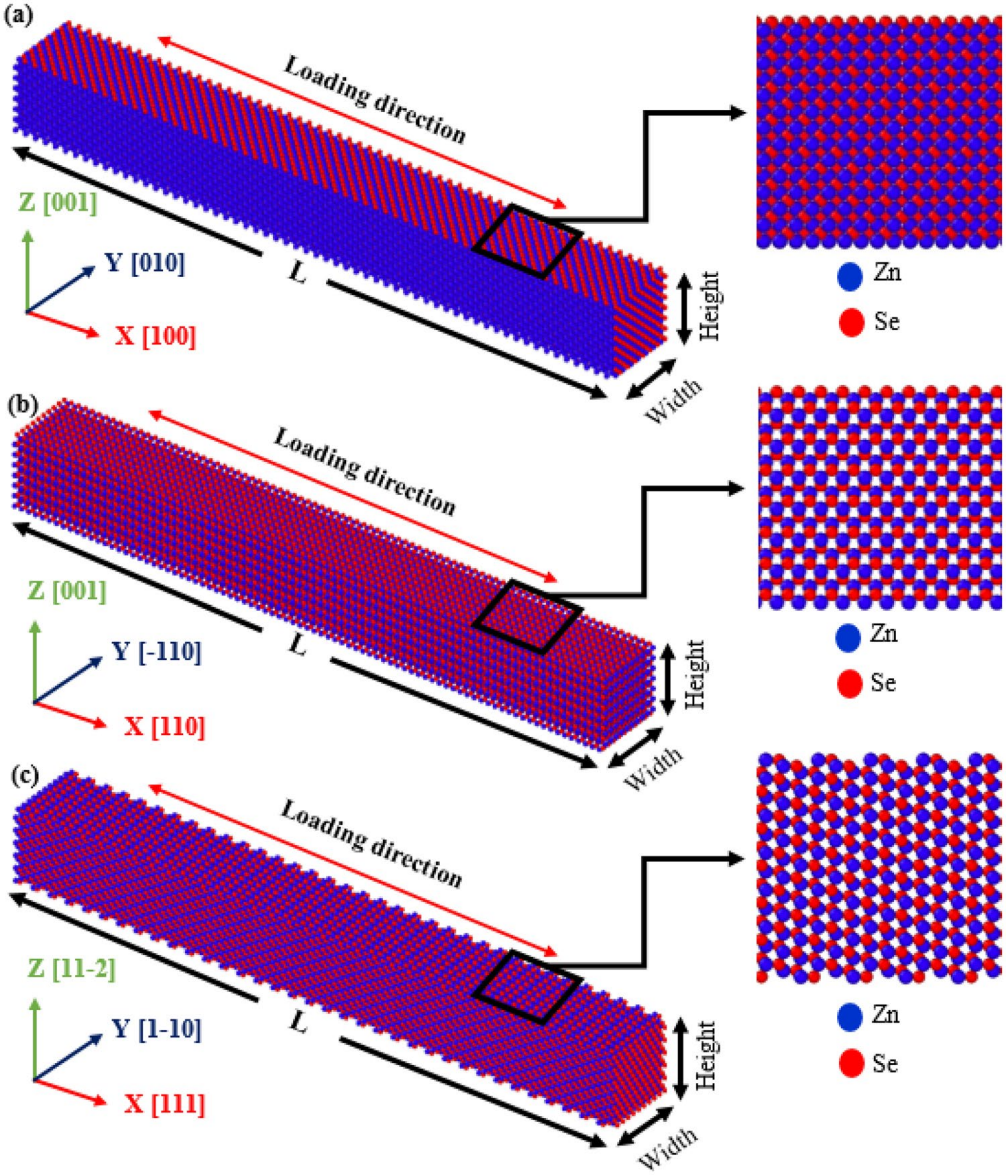
Initial structure of the (**a**) [100]-directed, (**b**) [110]-directed and (**c**) [111]-directed zinc-blende ZnSe NW with a dimension of ~ 34 nm × 3.97 nm × 3.97 nm. The cross-sectional area of all the NWS models is ~ 15.76 nm^2^.

The tensile mechanical characteristics and deformation mechanisms of zinc-blende ZnSe NW were explored using MD simulation using the LAMMPS program^48^. To integrate the usual Newton equations of motion in time, the Velocity Verlet approach with a time step of 1 fs was used. The Stillinger–Weber (SW) potential developed by Zhou et al.^47^ was used for defining the interatomic interactions between atoms of the Zn–Se, Zn–Zn, and Se–Se systems. The NW models are created by forming a rectangular box of zinc-blende ZnSe with a = 5.6676 Å as the lattice constant and then creating NWs from a defined rectangular pillar using the Atomsk tool^49^. The study considers loading along three crystal orientations: [100], [110], and [111]. We considered rectangular nanopillar structures consisting of ~ 23,520 to 23,800 atoms with a dimension of ~ (34.35 nm × 3.97 nm × 3.97 nm) by maintaining a length-to-width ratio of 8.57:1^16,50^. Figure 1 shows the [100], [110], and [111] crystal-oriented zinc-blende ZnSe NW models with a cross-sectional area of ~ 15.76 nm^2^. The tensile mechanical characteristics of the different crystal-oriented zinc-blende ZnSe NWs were examined in relation to different temperatures and strain rates. Temperatures were varied from 100 to 600 K, while strain rates varied from 1 × 10^8^ s^−1^ to 1 × 10^10^ s^−1^. To guarantee adequate space for the NWs to deform freely, a relatively large surface of 10 nm by 10 nm in the x–y plane was used. To further examine the impact of shape and diameter on the mechanical behavior of [111]-oriented ZnSe NWs, we also considered hexagonal structure in addition to square structure. The thickness and diameter for both shapes varied from ∼4 to ∼16 nm. Square and hexagonal-shaped ZnSe NWs with different thicknesses and diameters are shown in Fig. S1 (Supporting Information).

The conjugate gradient method was used to perform the energy minimization. After that, the system was relaxed in the NPT ensemble (constant atom number, pressure, and temperature) at a certain temperature with zero pressure applied in the axial direction for 40 ps. Finally, the system is thermally relaxed for 40 ps using a canonical (NVT) ensemble. In these procedures, a Nose–Hoover thermostat is used to control the temperature. The periodic boundary condition was used in the axial direction, while the fixed boundary conditions were used in the other two directions. We examined the energy conservation of zinc-blende ZnSe NWs in the NVE ensemble (constant atom number, volume, and energy) to verify the reliability of the time step used in our MD simulations. As demonstrated in Fig. S2a–c (Supporting Information), the total energy of NWs considered in this work ([111], [110], and [100]-oriented) remains almost constant over the NVE simulations, demonstrating that the time step used in our MD simulations is appropriate. Furthermore, the lattice constant of zinc-blende ZnSe NWs was discovered to be 5.6682 Å in the x direction, which agrees well with the experiments by Karzel et al.^51^ (5.6676 Å) and first-principles calculations by Okoye et al.^38^ (5.666 Å), Khenata et al.^52^ (5.624 Å), and Wang et al.^53^ (5.633 Å). This result indicates that the SW potential utilized in this work may adequately explain atom interactions in the ZnSe crystal. Moreover, to explore the deformation mechanisms, all visualizations are done with the OVITO package^54^.

After the energy minimization and structural relaxation, we employed uniaxial tensile loading along the length direction of the NWs at a constant strain rate of 10^9^ s^−1^. This is a favorable strain rate for MD simulations because of the computational restrictions, and it has been used effectively in several previous studies^16,50^. Because a considerably lower strain rate is being used experimentally, different strain rates were also considered to explore the influence of strain rate. In our models, the atomic stresses are estimated using the Virial stress theorem^55^, which is acquired as the arithmetic mean of the native stresses on all atoms and has the resulting expression:

1 $$\sigma_{virial} (r) = \frac{1}{\Omega }\sum\nolimits_{i} {\left[ {\left( { - m_{i} \dot{u}_{i} \otimes \dot{u}_{i} + \frac{1}{2}\sum\nolimits_{j \ne i} {r_{ij} \otimes f_{ij} } } \right)} \right]}$$

where Ω denotes the total volume of the NW, m_*i*_ denotes the mass of the atom *i*, ${\dot{\text{u}}}_{i}$ denotes the velocity component of atom *i*, $\otimes$ is the cross product, *r*_*ij*_ denotes the distance between atom *i* and *j* and *f*_*ij*_ is the force exerted by atom j on atom i.

## Results and discussion

In group II–VI and III–V binary semiconductor NW systems, due to the crystal polarity of the material and the combination of low energy and high symmetry of the [111] direction, the ZnSe NWs tend to grow in a zinc-blende cubic structure along the [111] direction^56^. However, in recent years, non-[111]-oriented semiconductor NWs have attracted increasing interest in terms of fundamental research and promising applications due to their outstanding crystal quality and distinctive physical properties^57–59^. Introducing numerous growth techniques, including chemical vapor deposition and the vapor–liquid–solid growth mode of low-pressure metal–organic vapor-phase epitaxy, the selective growth of stacking fault free ZnSe NWs along [100], [110], and [112] crystal orientations is reported^56,60^. By maintaining a cross connection among the NWs diameter, length, and growth temperatures, 99% of the total ZnSe NWs are shown to be grown either along [100], [110], [111], or [112] growth directions and are verified by photoluminescence spectroscopy, X-ray diffraction, and transmission electron microscopy^59,61^. Understanding the mechanical properties and deformation behavior of ZnSe NWs grown along the [100], [110], and [111] directions is thus critical for developing and optimizing their applications in various fields, such as nanoelectronics and nanoelectromechanical systems.

**Figure 2:**
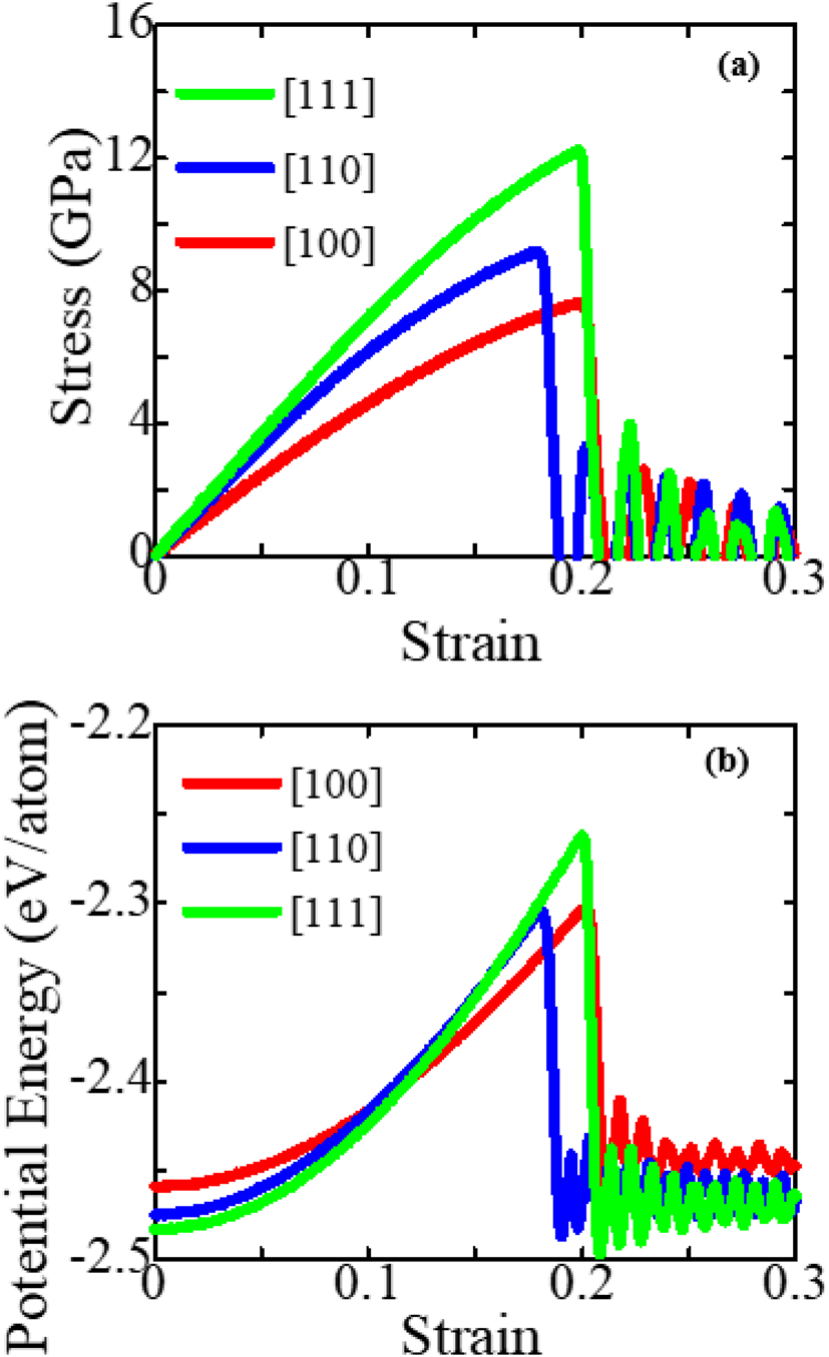
(**a**) Stress–strain response and (**b**) potential energy/atom of 15.76 nm^2^ zinc-blende ZnSe NWs for different crystal orientations at 300 K.

Firstly, we investigate the significance of crystal orientation on the stress–strain response behavior of the zinc-blende ZnSe NWs. Figure 2a reveals the room temperature stress–strain plots of a square cross-section zinc-blende ZnSe NWs subjected to uniaxial tensile loading at a strain rate of 10^9^ s^−1^ in the three crystal orientations [100], [110], and [111]. As can be observed, the curves are mostly made up of elastic and plastic phases. We can see from these curves that at 300 K, [111]-directed zinc-blende ZnSe has the highest fracture stress behavior, whereas the [100] loading direction has the lowest. Analogous types of orientation-dependent fracture stress behavior were also reported for CdSe, CdTe, InP, Ni-Co, and SiGe NWs^16–18,62,63^. Conversely, the [100]-oriented zinc-blende ZnSe displays more significant fracture strain than the other two crystal directions. The reason behind this greater fracture strain required to deform the [100]-oriented NW will be discussed later in this section. The area underneath the stress–strain behavior can be used to calculate fracture toughness, which is the extent of energy required prior to failure^16^. The exact value of the fracture toughness of bulk ZnSe might differ depending on several factors, including grain size, crystal structure, and the presence of defects or impurities. On the other hand, the fracture toughness of ZnSe NWs can depend on the size and shape of the NW, the crystalline direction, and the orientation of the material with respect to the applied stress^16,50^. This study found that due to the favorable crystal structure, [111]-directed ZnSe NW has the highest fracture toughness, while the [100] loading direction has the lowest value. Afterward, using a graphical technique, we calculated the elastic modulus for all NWs systems based on the slope of the stress–strain curve in the linear region (up to ≤ 1% strain). The small strain region ensures that the configuration follows Hook's law and that linear elastic destruction is achieved. The estimated elastic modulus for three crystal orientations of zinc-blende ZnSe NWs at 300 K with a thickness of ~ 4 nm is found to be 50.08 GPa, 72.03 GPa, and 74.46 GPa, respectively, along the [100], [110], and [111] orientations. The [111] direction has the maximum elastic modulus of ~ 74.46 GPa, indicating that the ZnSe NW has high stiffness and is difficult to deform. In the [111] loading direction, the maximum fracture stress and elastic modulus are caused by the lowest surface energy of the materials. Previous research has shown that the [111] crystal orientation has the longest surface atomic distance and thus the smallest concentration of surface atoms, allowing it to have the lowest surface energy, followed by the [110] and [100] crystal orientations^64,65^. Hence, when a tensile force is applied in the [111] direction, the [111] surface with the lowest energy has the most significant capacity to resist fracture^64,65^. To explain the effect of NW orientations on the stress–strain performance, we consider the zoomed-in view of the surface of the zinc-blende ZnSe NWs along the three orientations, as shown in Fig. 1a–c. From the zoomed-in view, it can be seen that the [100]-directed NW surface shows a very dense structure compared to the [110] and [111]-directed NW surfaces. Therefore, the surface atomic distance of the [100]-oriented NW will show a smaller value than the other two directions. Moreover, to signify the surface atomic distance of different NWs, we have calculated the potential energy per atom of [111], [110], and [100]-oriented ZnSe NWs, which are shown in Fig. 2b. From the figure, it has been noticed that as [111]-oriented NW shows very low surface density compared to the other two directions, the potential energy per atom at zero strain shows a lower value for that direction, which signifies its superior structural stability and strength, including fracture strength and elastic modulus, compared to the other directions.

**Figure 3:**
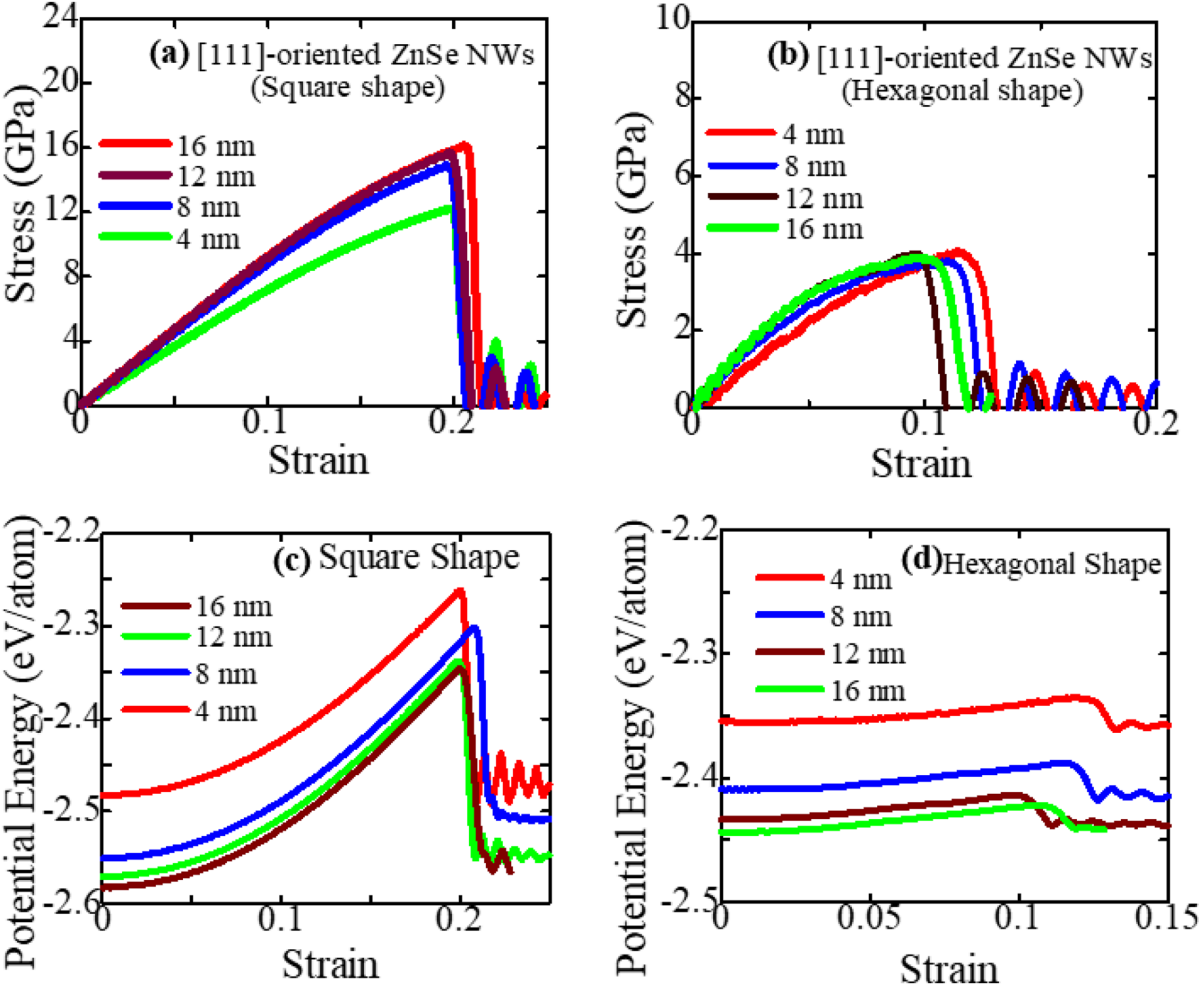
Effect of thickness or diameter on the stress–strain response curves of [111]-oriented zinc-blende ZnSe NWs in (**a**) square shape and (**b**) hexagonal shape at 300 K. Thickness-dependent potential energy/atom of [111]-oriented zinc-blende ZnSe NWs in (**c**) square and (**d**) hexagonal shapes at 300 K.

Moreover, zinc-blende ZnSe NWs with [111] orientation can be grown in different shapes with thickness ranges from 8 to 30 nm or above^1,2,56,60,61,66^ using molecular-beam epitaxy and the vapor–liquid–solid mechanisms. To make the next generation of nanotechnology more enduring and lightweight, researchers and experimentalists are also working hard to develop smaller diameter NWs^2^. Hence, in this study, we have also studied the mechanical behavior such as fracture stress and elastic modulus of [111]-oriented square and hexagonal-shaped ZnSe NWs with thickness varying from ~ 4 to ~ 16 nm. The stress–strain relationship of square and hexagonal-shaped ZnSe NWs at 300 K for four different thicknesses or diameters throughout the tensile process is shown in Fig. 3a,b, respectively. The results show that for both types of shapes, the ultimate tensile strength increases as the thickness or diameter of the NWs increases; this outcome is consistent with other varieties of semiconductor NWs^5,16,67–69^. In particular, as the thickness increases from ~ 4 to ~ 16 nm, the ultimate tensile strength increases from 12.24 to 16.12 GPa for square-shaped ZnSe NWs. On the other hand, the ultimate tensile strength increases from 3.81 to 4.08 GPa for hexagonal-shaped ZnSe NWs with the increase in thickness from ~ 4 to ~ 16 nm. For both square and hexagonal-shaped ZnSe NWs with different thicknesses, the elastic moduli are then determined using stress–strain curves with (strain ≤ 1%) linear regression and are shown in Table 1. We compared our computed elastic modulus of zinc-blende ZnSe NWs at 300 K with previous published studies^52,53,70,71^, as shown in Table 1, to validate the interatomic potential and computational approach employed in this work. The outcomes show the possibility of correctly predicting the mechanical characteristics of the ZnSe nanostructures using the SW interatomic potential. Previous research discovered that elasticity is size-dependent^2^ and is restricted to NWs with diameters less than 20 nm. Therefore, our work also shows a similar size dependency, which confirms our investigation. Most importantly, the square-shaped ZnSe NWs for all considered thicknesses show greater mechanical behavior than hexagonal-shaped ZnSe NWs. The potential energy per atom at zero strain is shown in Fig. 3c,d, which explains why this variation occurs. For similar thickness or diameter, the potential energy per atom at zero strain shows greater negativity for square-shaped NWs compared to hexagonal shapes; verify our calculations. Moreover, since the elastic modulus and fracture stress both rise with increasing thickness at a certain temperature, it is evident that the thickness of the ZnSe NW has a substantial impact on its tensile mechanical characteristics. The de-cohesion effect of the surface atoms, which is caused by the large surface area to volume ratio of NWs, is the cause of their size-dependent mechanical characteristics^68,72,73^. Furthermore, mechanical behavior variation is caused by NWs lattice defects, which are primarily caused by surface atoms. The surface atoms will move inward to the core as the surrounding coordinates decrease, resulting in lattice defects^16^. However, NWs with greater thickness are less susceptible to surface defects compared to NWs with smaller thickness, and hence, when the thickness increases, mechanical characteristics such as the elastic modulus and fracture stress of ZnSe NW show an increasing nature.

**Table 1:** Calculated elastic modulus of [111]-oriented zinc-blende ZnSe NWs at 300 K.

Diameter/thickness (nm)	Elastic modulus (GPa) found in this study		Elastic modulus (GPa) found in literatures
	Square shape NWs	Hexagonal shape NWs	
∼4	74.46	47.59	81.0^52^, 82.8^53^, 85.9^70^, 88.8^71^
∼8	89.49	60.35	
∼12	92.44	67.12	
∼16	94.75	67.92	

**Figure 4:**
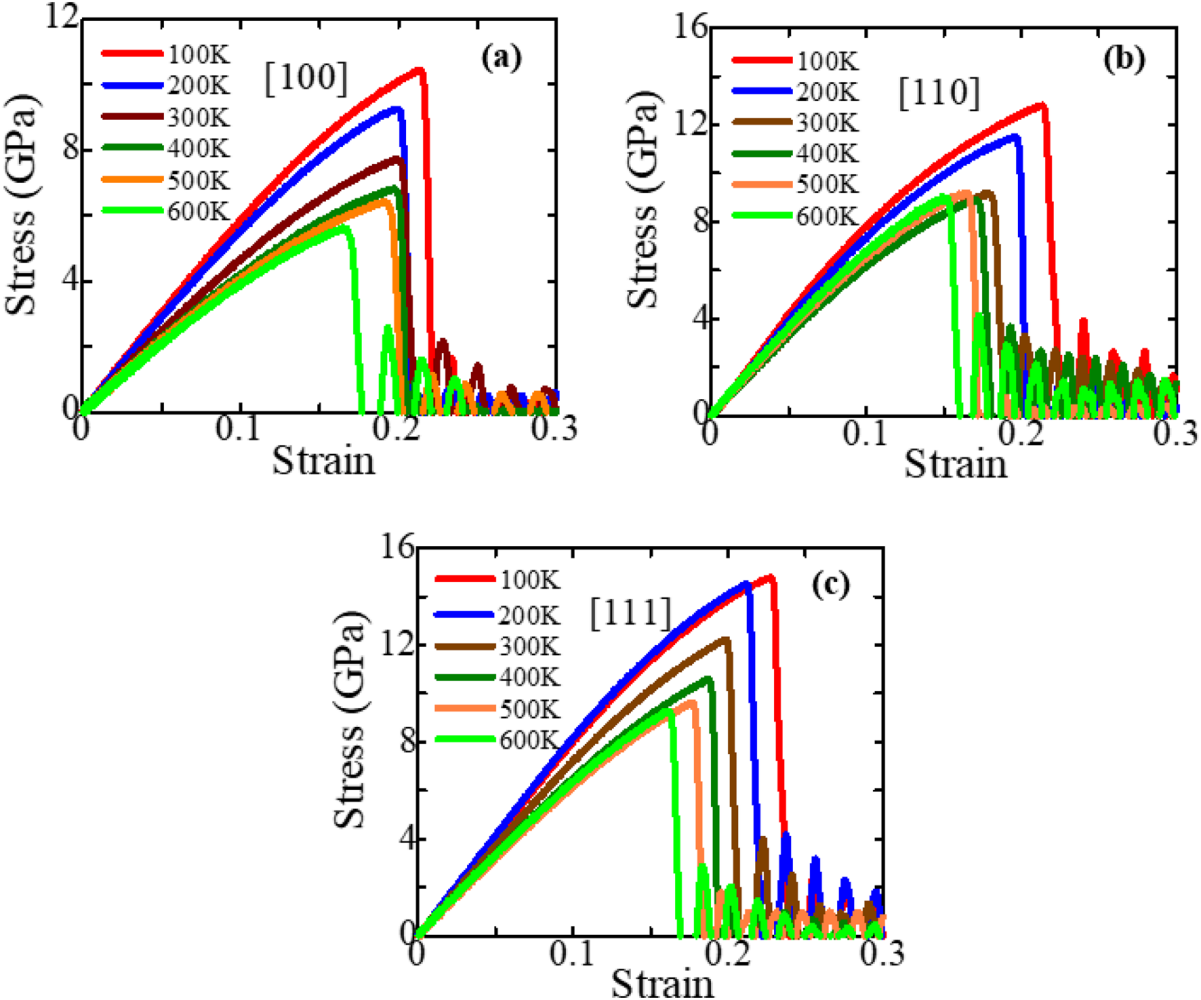
Stress–strain response of (**a**) [100], (**b**) [110], and (**c**) [111]-oriented 15.76 nm^2^ zinc-blende ZnSe NWs at different temperatures.

**Figure 5:**
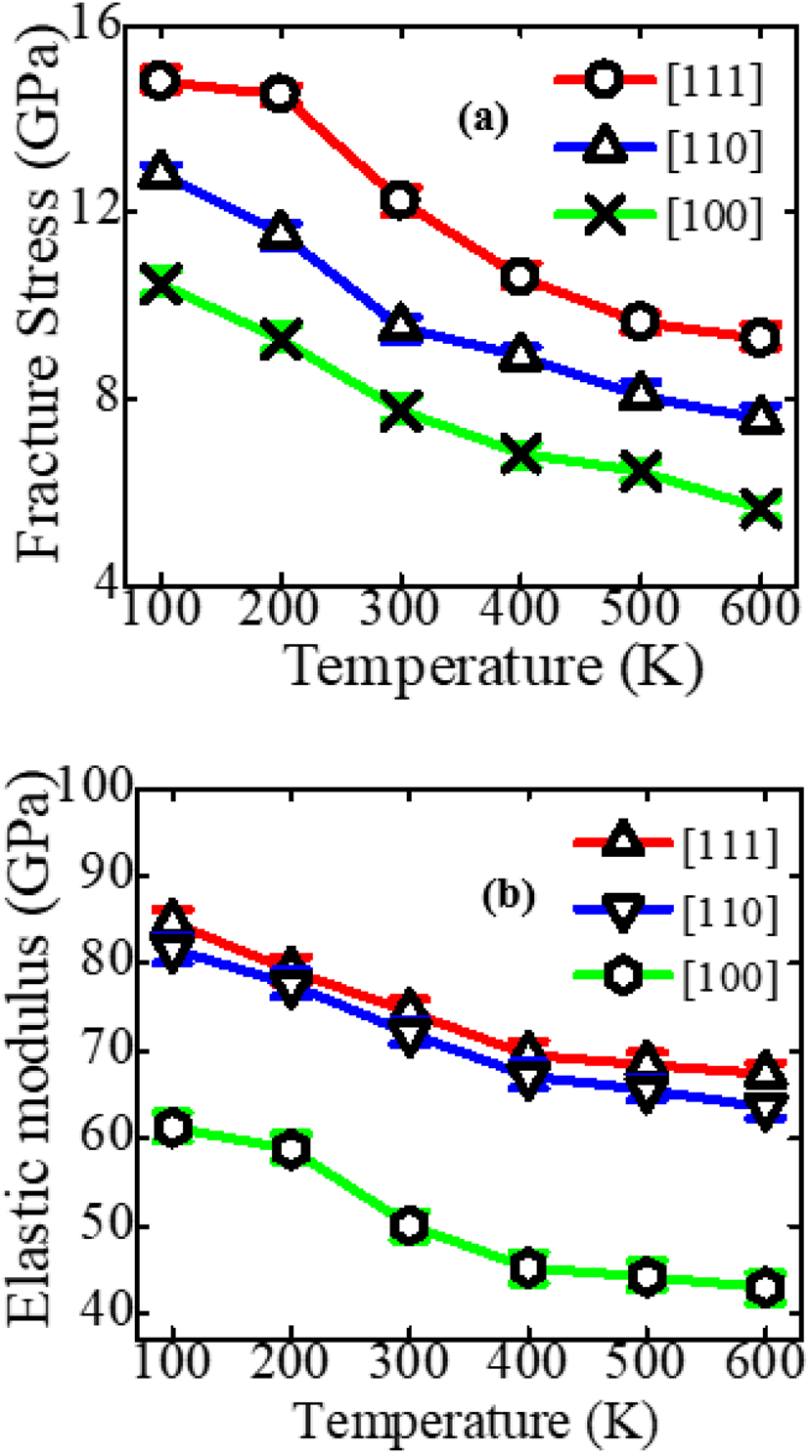
(**a**) Fracture stress and (**b**) Elastic modulus of 15.76 nm^2^ zinc-blende ZnSe NWs for different crystal loading directions with different temperatures.

Temperature is a powerful external parameter that can significantly affect the NW’s mechanical behavior. The brittle or ductile behavior of semiconductor NWs can be significantly affected by the temperature and loading conditions^5,16,67–69,74^. At high temperatures, the thermal energy can cause the bonds between atoms to weaken, making the material more prone to plastic deformation and resulting in a more ductile response. On the other hand, low temperatures can make the material more brittle, with a lower amount of plastic deformation before fracture. Here, we conducted several MD simulations for numerous temperatures to explore the temperature dependence of the mechanical behavior and deformation mechanism of zinc-blende ZnSe NWs. Figure 4a–c depicts the stress–strain plots of zinc-blende ZnSe NWs for various crystal orientations at temperatures ranging from 100 to 600 K. As the figures demonstrate, when the temperature is increased from 100 to 600 K, the fracture stress and the fracture strain decrease sharply. Furthermore, the peak in the stress versus strain curve becomes smooth rather than abrupt, and the stress variation in the plastic distortion part turns is minimal. Therefore, zinc-blende ZnSe NW exhibits conventional brittle behavior and no temperature for the transition from brittle to ductile is identified. The stress–strain curves at various temperatures are used to calculate the ultimate fracture strength and elastic modulus (shown in Fig. 5a,b). At a temperature of 100 K, the ZnSe NW has a maximum fracture stress of ~ 10.46 GPa, ~ 12.82 GPa, and ~ 14.79 GPa, along the [100], [110], and [111] directions, respectively. The fracture stress drops to ~ 5.65 GPa, ~ 7.56 GPa, and ~ 9.29 GPa along the [100], [110], and [111] directions, respectively, as soon as the temperature rises to 600 K. The fracture stress of the zinc-blende ZnSe NW decreases by 45.98%, 41.02%, and 37.24% along the three directions when the temperature is increased to 600 K. Like fracture stress, the estimated elastic modulus also shows a decreasing trend with increasing temperature. As shown in Fig. 5b, when the temperature increases from 100 to 600 K, the elastic modulus decreases from ~ 61.27 to ~ 42.99 GPa, ~ 81.67 to ~ 63.56 GPa, and ~ 84.69 to ~ 67.37 GPa along the [100], [110], and [111] directions, respectively. The elastic modulus of the zinc-blende ZnSe NW decreases by 29.84%, 22.16%, and 20.45% along the three directions when the temperature is raised to 600 K. Because of the different effects, such as thermal expansion, greater atomic mobility, and fast diffusion of free volume at high temperatures, the elastic modulus decreases as temperature rises. Moreover, Fig. 5a shows that the rate of decrease in fracture stress with temperature is most significant for the [100] orientation and lowest for the [111] orientation. Similarly, for the three crystal orientations, the rate of reduction in elastic modulus with temperature is also greatest for the [100] orientation and lowest for the [111] orientation. In a later section, we will look at the deformation mechanisms of different crystal-oriented NWs that cause this reducing trend variation. The potential energy is a valid measure to quantify the reducing trend of mechanical strength for different orientations, which we have already shown and explained in Fig. 2b. Furthermore, the total energy per atom prior to strain can be used to determine why mechanical strength decreases with increasing temperature. Hence, the total energy per atom curves of [111], [110], and [100]-oriented zinc-blende ZnSe NWs for six different temperatures before tensile deformation are presented in Fig. S2a–c (Supporting Information). Before applying tensile force, 40,000 MD steps are used for equilibration. However, the figure shows only the first 20,000 MD steps for all considered temperatures. As can be seen from Fig. S2, zinc-blende ZnSe NWs for all different crystal orientations show stable behavior after 1000 MD steps at all temperatures. Furthermore, as the kinetic energy of atoms increases with temperature, so does our calculated total energy per atom curve, with a higher value found for 600 K for all orientations, indicating that the structures are less stable than for zinc-blende ZnSe NWs at 100 K. Hence, lower strain energy is sufficient to deform the NW when the temperature increases.

The radial distribution function (RDF), g(r), shows how the density of neighboring atoms around a particular atom varies as a function of distance. The distribution around that atom is computed by the distance between the specific target atom and neighboring atoms^17,75^. The temperature has a significant effect on the g(r) function. Due to the increase in temperature, there is substantial lattice vibrations in the atomic arrangements, and the separation distances of various pairs formed in the systems also show changing behavior. We have estimated the RDF at three different temperatures to qualitatively elucidate the temperature-dependent mechanical performance. Figure S3a–c (Supporting Information) depicts the temperature-dependent RDFs of Zn–Zn, Zn–Se, and Se–Se pairs of the ZnSe system. The g(r) peaks are extremely tall and thin at a low temperature of 100 K, indicating a solid crystal structure that is well-ordered and compact. However, the particles become more energetic when the temperature rises and oscillate from their equilibrium position. As a result, the height of the g(r) peaks drops. At the same time, the breadth expands, indicating that the number of ordered crystal structures reduces while the number of disordered structures increases as temperature increases. As a result, when the structure is at a higher temperature, a smaller amount of uniaxial force is required to break it than when it is at a lower temperature. This form of RDF function with increasing temperature thus denotes the cause of mechanical strength loss at high temperatures, which is consistent with previous research^17^.

**Figure 6:**
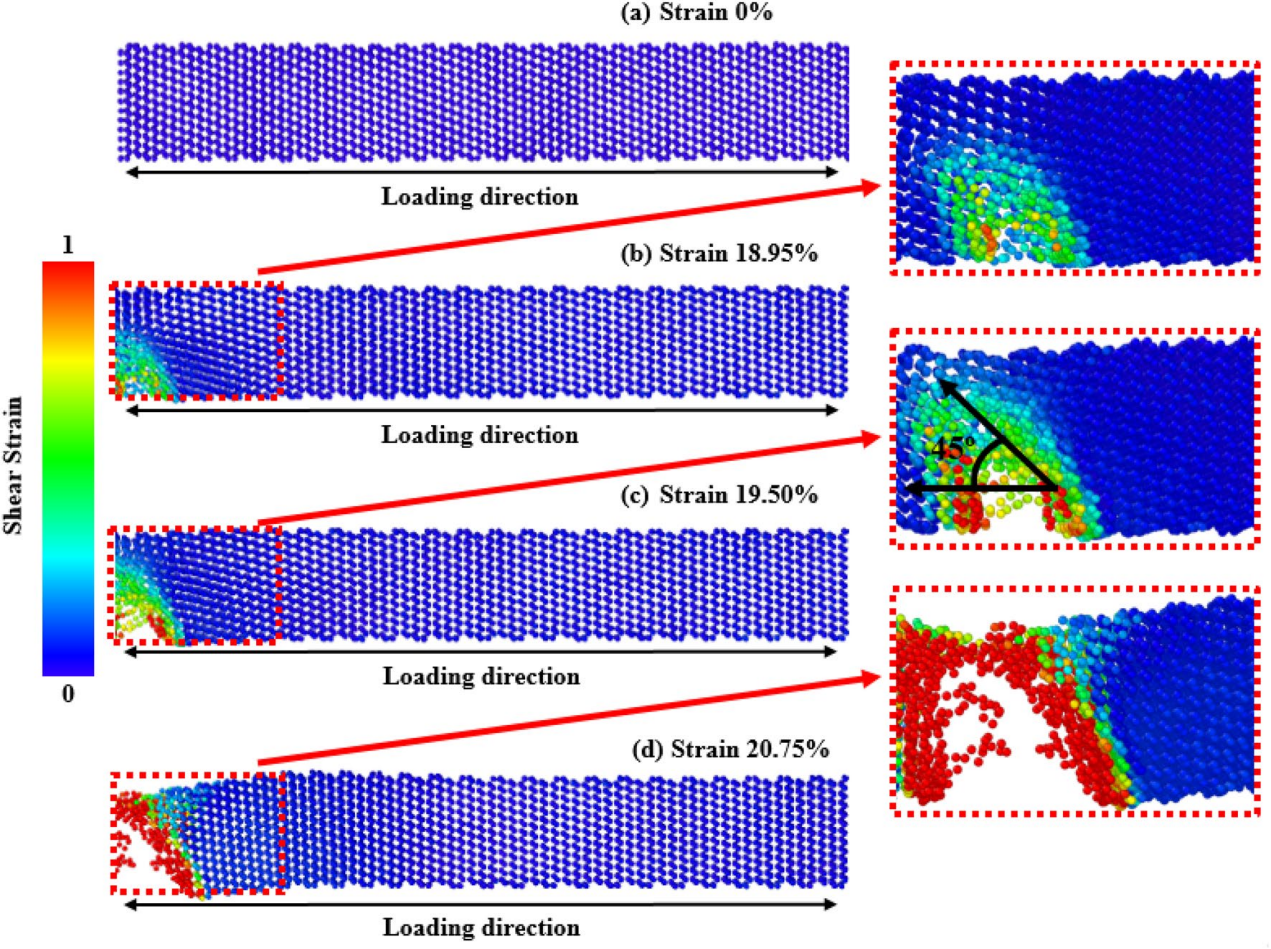
Tensile deformation profiles of [111] directed 15.76 nm^2^ zinc-blende ZnSe NWs for various strain levels at a temperature of 300 K. Dislocation slipping induced cleavage plane along an angle of 45° with the applied tension is shown in the zoomed-in view.

**Figure 7:**
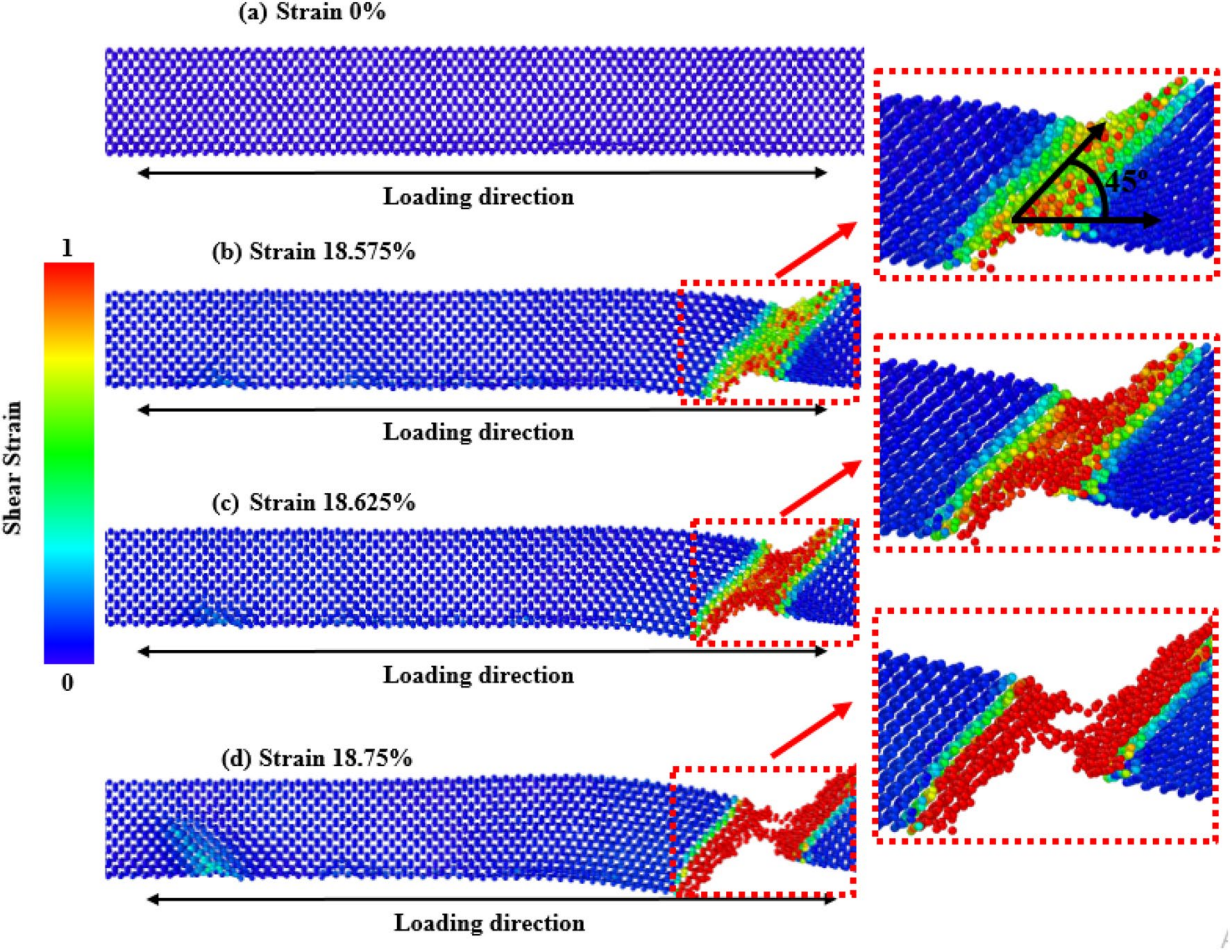
Tensile deformation profiles of [110] directed 15.76 nm^2^ zinc-blende ZnSe NWs for various strain levels at 300 K. Dislocation slipping induced cleavage plane along an angle of 45° with the applied tension is shown in the zoomed-in view.

**Figure 8:**
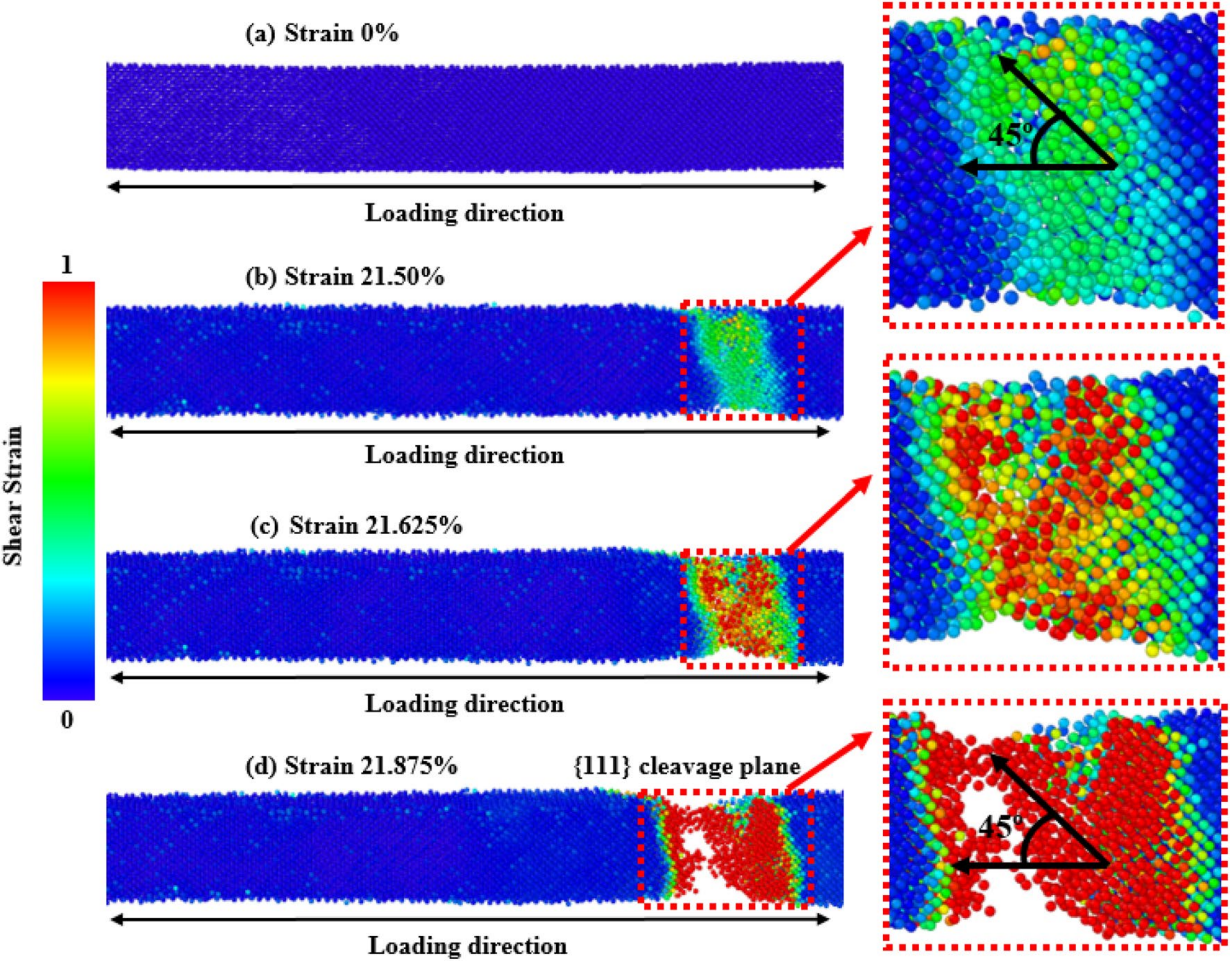
Tensile deformation profiles of [100] directed 15.76 nm^2^ zinc-blende ZnSe NWs for various strain levels at 300 K. The zoomed-in view shows the dislocation slipping induced {111} cleavage plane along an angle of 45° with the applied tension.

Deformation mechanisms at different strain values can be used for quantitative and qualitative explanations of mechanical behavior^76–79^. Firstly, to explore the effects of crystal orientations, the stress propagation and deformation pattern of ZnSe NWs at room temperature for three different orientations ([111], [110], and [100]) are shown in Figs. 6, 7, and 8, respectively. According to the findings, the crack initiation of [100]-oriented ZnSe NWs (shown in Fig. 8) starts at a higher strain value compared to that of [111] and [110]-oriented ZnSe NWs, which proves our finding of Fig. 2a. Now the question arises: although the developed stress along the [100] orientation is lower compared to the [111] and [110] orientations, why does this orientation show a higher strain during its deformation? We have already explained that the surface energy of [111]-oriented ZnSe NWs is lower compared to [110] and [100] orientations, and hence [111] exhibits better fracture stress as well as elastic modulus. Nevertheless, the atomic distance between the atoms along the [111] direction (shown in Fig. 1c) is large compared to the [100] direction, and hence, a lesser extent of uniaxial tensile strain compared to the [100] direction facilitates the crack initiation. Finally, the structure deforms in a very short time. Furthermore, it has been reported that at room temperature or lower temperatures, due to its fully activated nature over other planes, the {111} plane can dominate the cleavage nucleation when a force is applied to its direction. Similar results for the mechanical properties of [111]-directed NWs were also reported for CdSe, CdTe, and InP NWs^16,50,62^.

Using a similar reasoning as above, the surface energy of [100]-oriented ZnSe NWs is very high compared to the [111] and [110] directions, and hence [100] shows lower fracture stress as well as elastic modulus. Here, as the atomic distance between the atoms along the [100] direction is smaller than the other orientations (shown in Fig. 1a), the nucleation of a crack along the {100} plane would demand a considerable amount of energy. Moreover, as the bonding shows strong rigidity, it needs a more significant tensile strain to deform the structure. As shown in Fig. 8, it can be noticed that at room temperature, [100]-directed NWs need a large strain value of 21.875% to initiate the structure's deformation compared to other orientations owing to the dominance of the smaller interatomic distance, producing an enormous attractive electrostatic force amid the planes^50^. At room temperature along the [100] orientation, due to the dominance of its smaller interplanar distance, the {100} plane was not fully activated, and hence the {111} cleavage plane activated with the applied tension and produced the result of lower fracture stress with higher strain. Similar results along [100]-oriented NWs mechanical behavior can also be found in the literature for CdTe and SiGe ZB NWs mechanical properties^62,67^. Moreover, for zinc-blende NWs, the principal source of native deformations that create NW collapse is the nucleation and transmission of dislocations through partial slip, full slip, or twinning. As the energy barrier for the nucleation of dislocation is lower at edges than on faces or in the bulk, the disruption originates from one of the edges of the NW, circulates through the cross-section, and finally forms one of the local deformations. For [110] and [111]-oriented NWs, it has been noticed that dislocation slip is initiated at the left or right edges of the structures. One type of cleavage plane creates a deformation angle of 45° with the direction of the tensile force.

**Figure 9:**
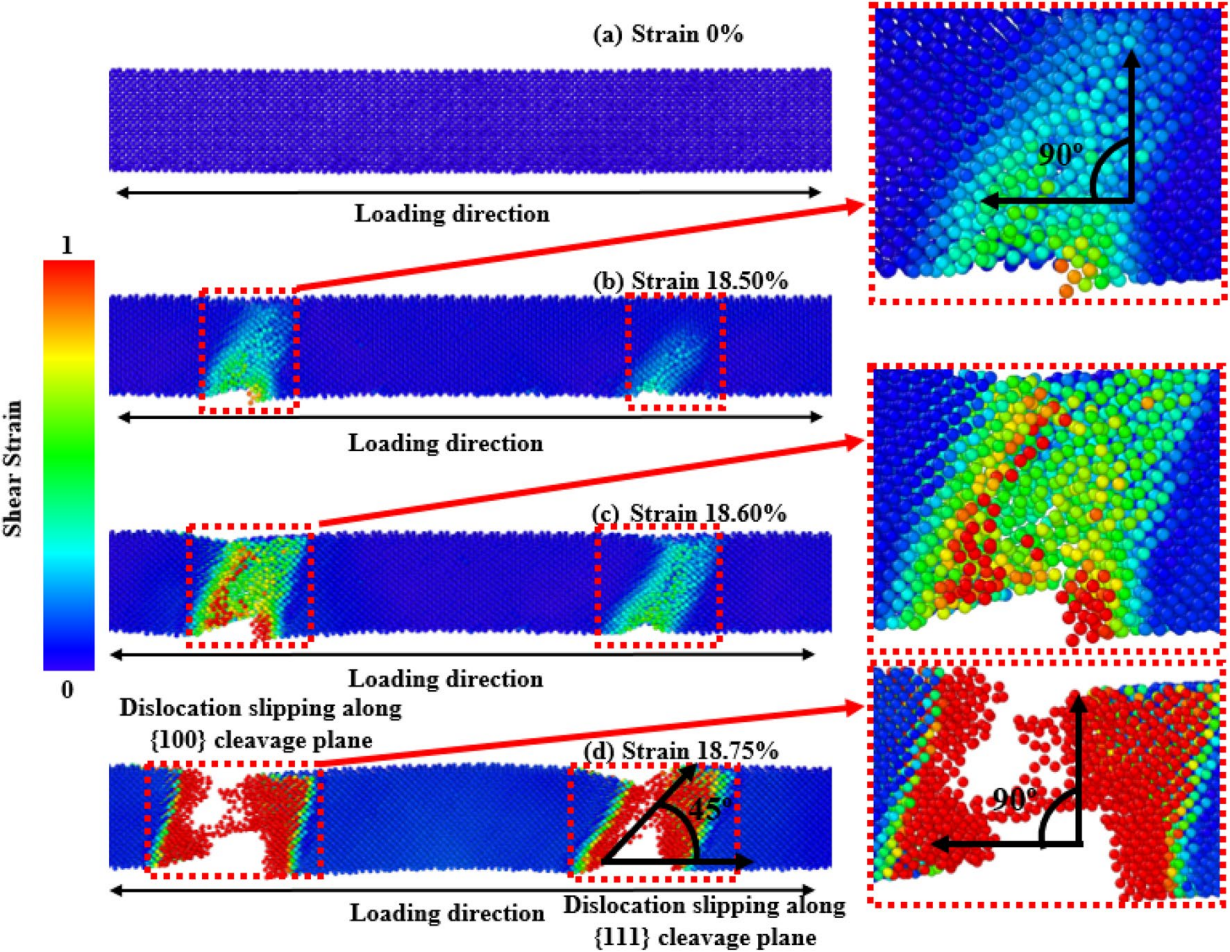
Tensile deformation profiles of [100] directed 15.76 nm^2^ zinc-blende ZnSe NW for various strain levels at 600 K. The zoomed-in view shows the dislocation slipping induced {100} cleavage plane along an angle of 90° with the applied tension.

As with the increase in temperature, all the different orientations show a reducing trend in mechanical behavior, and especially the [100]-oriented ZnSe NW shows a greater reduction. Here, we have only considered the stress propagation and deformation pattern of [100]-oriented ZnSe at 600 K to understand the effects of increasing temperatures. The deformation profiles of [100]-oriented ZnSe NWs at 600 K are depicted in Fig. 9. The fracturing process of [100]-oriented ZnSe NW began with the initiation of a crack at 21.50% strain and ended with the ultimate failure at 21.875% strain at 300 K temperature. When the structure is at 600 K, the crack initiates with a strain value of 18.50%. It happens because the zinc-blende ZnSe crystal's atomic linkages experience more thermal fluctuations at higher temperatures, which eventually cause the chemical bonds to weaken^16,50^. Moreover, as the influence of thermal vibrations is very high at higher temperatures, a minimal strain is sufficient to disrupt the bond and create the void, destroying the pristine NWs^16,50^. After fracture initiation, the crack begins to branch, and the major atomic displacement occurs in the loading direction. The initial crack generation and eventual collapse happens at nearly the same strain level, suggesting a brittle failure manner. At 300 K temperature we noticed that at 21.625% strain, fractures began to form at a single location of the NWs, (as illustrated by red blocks in Fig. 8), and bonds began to break at around 21.875% strain. This happened because the structure only experiences a modest thermal vibration at low temperatures, which prevents the distortion and stress from rapidly spreading from the initial bond rupture in the NW to the entire arrangement and the lack of activation in the {100} plane. However, when the temperature is considerably high and increased to 600 K, the magnitude and the rate of atomic vibration increases, and atoms can travel more effortlessly from their equilibrium location, which increases the mean interatomic distance and activates the {100} plane strongly. As a result, at a greater temperature, the comparative importance of interplanar distance reduces. Cracks initiate at two different places (denoted by red square-shaped dotted blocks) due to the combined effects of the {111} plane (making an angle of 45° with the direction of tension) and the {100} plane (making an angle of 90° with the direction of tension)^16,50^. Because of the activation of two types of planes as the temperature increases, the fracture stress and elastic modulus of zinc-blende ZnSe NWs rapidly decreases, and the NWs collapse at very low strain levels.

It is worth noting that MD simulations are computationally intensive, and simulations at lower strain rates need very long computational times^50,80^. Therefore, in recent times, most of the tensile tests using MD simulation have been done at strain rates of 10^8^ to 10^10^ s^−1^, which are very popular, computationally efficient, and can predict the experimental description of the considered systems^2,5,16,67^. Besides, high strain rates in MD simulations allow to study the mechanical behavior of materials under extreme loading conditions that may be difficult or impossible to obtain experimentally^80–82^. It has been reported that due to the variation of strain rates from low to high, there is a change mainly in the plastic phases of the stress–strain curves, which is responsible for the variation of ultimate tensile strength and toughness. In the plastic phase of the NWs, different failure mechanisms, including partial slipping, dislocation slipping, twinning, reorientation, necking, amorphization, etc., are thus induced with varying strain rates^83^. Moreover, the difference in strain rates between the simulations and experiments can indeed affect the observed brittle or ductile behavior of the semiconductor NWs. In general, smaller strain rates can lead to more gradual and plastic deformation, which may result in a more ductile response characterized by a larger amount of plastic deformation before failure. However, the smaller strain rate-based brittle or ductile behavior of semiconductor NWs can be influenced by the size and shape of the NWs, crystal structure and orientation, the presence of defects and impurities, and the temperature and loading conditions.

**Figure 10:**
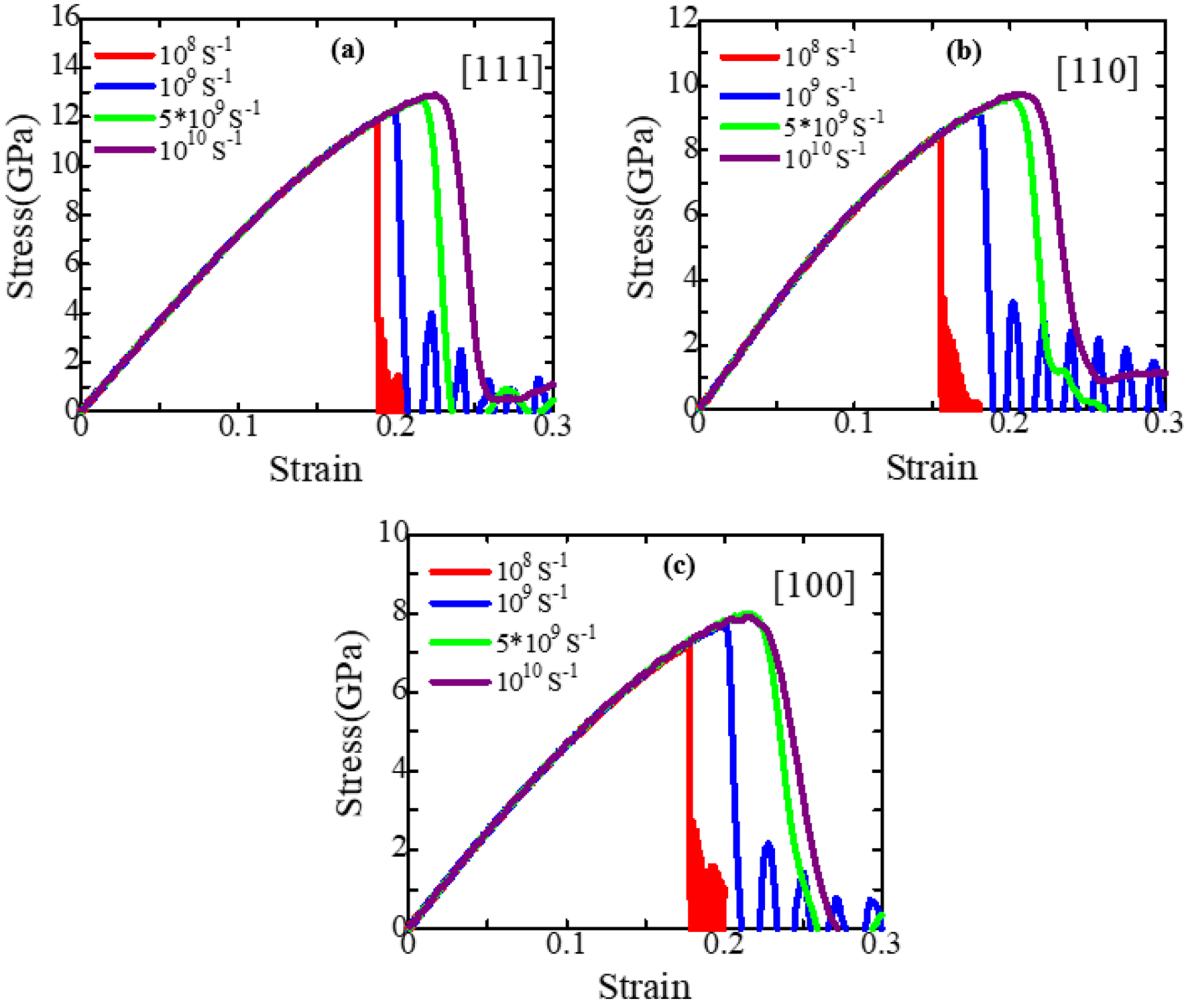
Stress–strain relationship of (**a**) [111], (**b**) [110], and (**c**) [100]-oriented ZnSe NWs for different strain rates at 300 K.

In this study, we have considered strain rates from 10^8^ s^−1^ to 10^10^ s^−1^ at 300 K to explore their consequences on the mechanical behavior of ZnSe NWs for three crystal orientations. Figure 10 shows the effects of numerous strain rates varying from 10^8^ s^−1^ to 10^10^ s^−1^ on the stress–strain performance of [111], [110], and [100]-oriented zinc-blende ZnSe with a cross-sectional area of ~ 15.76 nm^2^. Before reaching the maximum stress point, the material's stress–strain performance is independent of strain rates, meaning that strain rates do not influence elastic modulus. However, for zinc-blende ZnSe NWs, when the strain rate declines, both the fracture stress and fracture strain drop. This drop results from temperature fluctuation and stress relaxation being favored at a slower strain rate because the atoms have more time to respond. As a result, the bond breaks down earlier because the atoms can cross the energy barrier at a lower tension. On the other hand, insufficient time to relax at a higher strain rate creates rapid fluctuations in atoms, which cause cracks to start to nucleate from different regions of the NW simultaneously and propagate instantly. Therefore, slight distortions in the stress–strain plot corresponding to a strain rate of 10^10^ s^−1^ are found. Moreover, when the strain rate increases, both rupture strength and strain rise, which is consistent with prior observations of comparable zinc-blende NWs^16,50,84^. Table 2 lists the failure stress and elastic modulus parameters obtained from Fig. 10. Because the slopes of the stress–strain curves in the elastic area during deformation coincide for different strain rates, the elastic modulus is determined to be strain rate independent. The relationship between the ultimate fracture strength of zinc-blende ZnSe NW along a particular crystal orientation and the strain rate can be related by the Arrhenius equation of^77^:

2 $$\dot{\varepsilon } = {\text{A}}\sigma^{\frac{1}{m}} exp\left( { - \frac{Q}{{{\text{RT}}}}} \right)$$

where $\dot{\varepsilon }$ signifies the strain rate, *σ* signifies the fracture strength, *Q* signifies the activation energy, R is the universal gas constant, T is the deformation temperature, *m* is the strain-rate sensitivity, and A is a constant. By using natural logarithms on both sides and assuming that the temperature remains constant throughout the deformation, Eq. (2) can be further simplified as:

3 $$\ln \dot{\varepsilon } = \ln \;(A) + \frac{1}{m}\ln \;(\sigma ) - \frac{Q}{RT}$$

Using the slopes of ln(*σ*) and ln($\dot{\varepsilon }$), the strain-rate sensitivity *m* along the different crystal orientations can be obtained using the following relationship:

4 $$m = \frac{\partial \ln (\sigma )}{{\partial \ln (\dot{\varepsilon })}}$$

**Figure 11:**
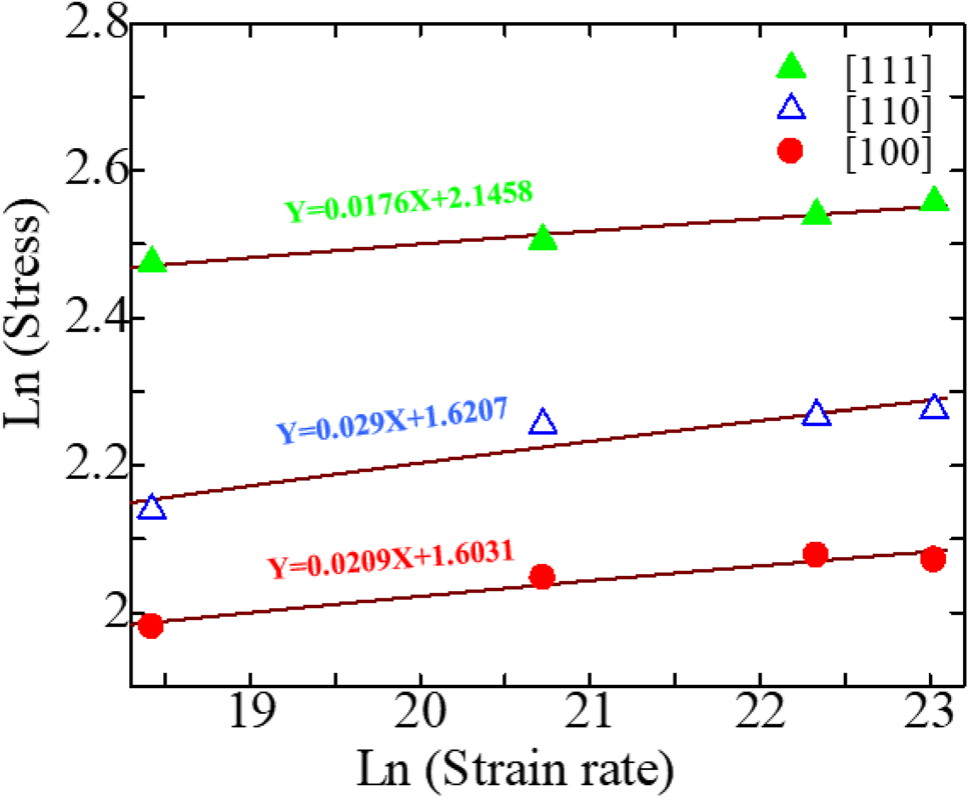
The strain-rate sensitivity of zinc-blende ZnSe NWs along three crystal orientations at 300 K.

Here, in this work, the strain-rate sensitivity m for [111], [110], and [100] orientations of zinc-blende ZnSe NW was found to be 0.0176, 0.029, and 0.0209, respectively; as shown in Fig. 11. Hence, the strain rate has a crucial effect on the [110]-oriented zinc-blende ZnSe NW. This high sensitivity along the [110] orientation may have occurred due to its deformation mechanisms.

**Table 2:** Calculated room temperature fracture stress and elastic modulus of three different crystal-oriented zinc-blende ZnSe NWs at different strain rates.

Strain rate (s^−1^)	Fracture strength (GPa)		
	[100]	[110]	[111]
1 × 10^8^	7.25	8.49	11.87
1 × 10^9^	7.75	9.55	12.24
5 × 10^9^	7.99	9.65	12.66
1 × 10^10^	7.94	9.73	12.89

Strain rate (s^−1^)	Elastic modulus (GPa)		
	[100]	[110]	[111]
1 × 10^8^	50.20	72.01	74.20
1 × 10^9^	50.08	72.03	74.46
5 × 10^9^	50.30	72.20	74.52
1 × 10^10^	50.40	72.37	74.64

**Figure 12:**
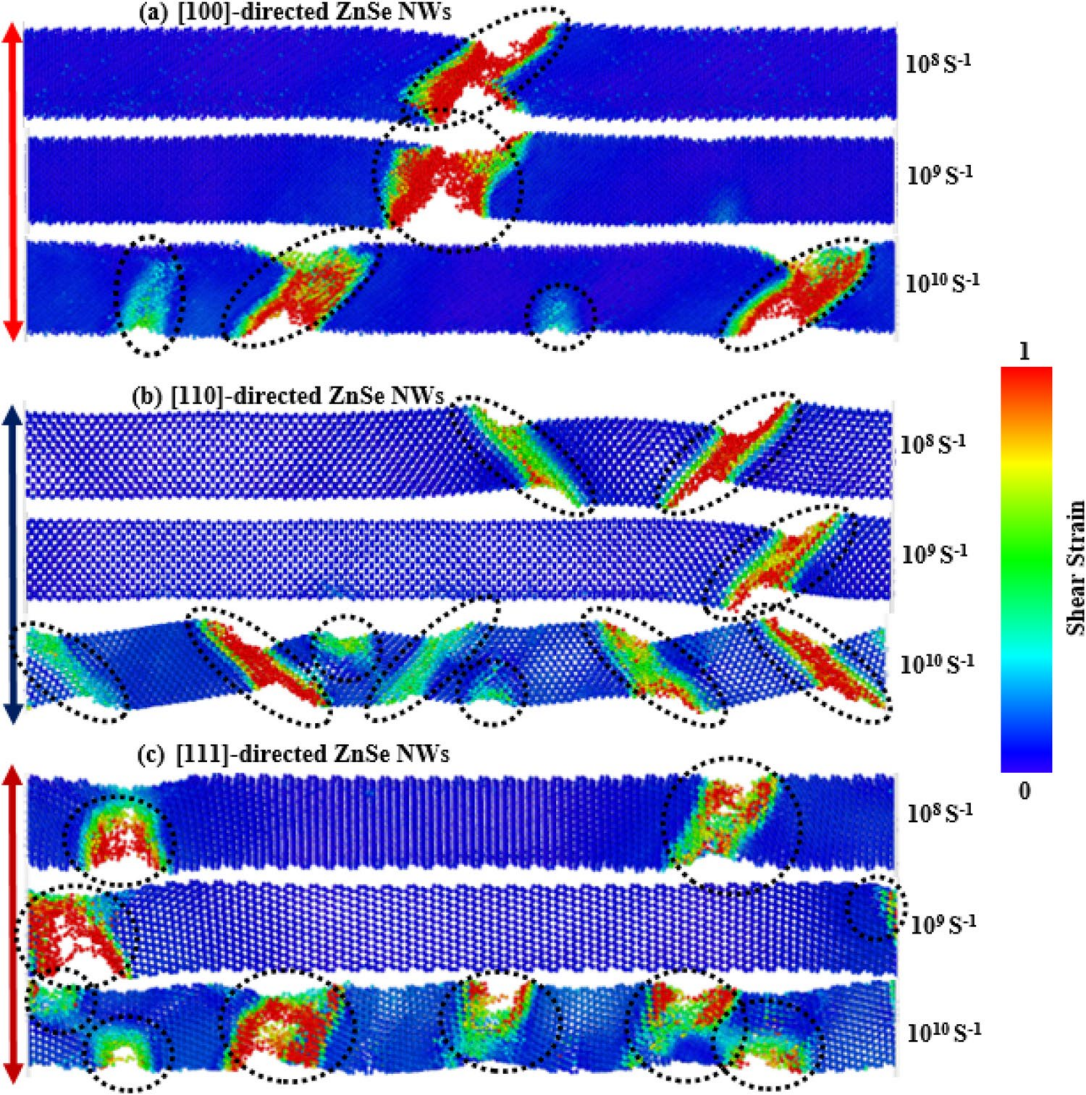
Variation in the deformation mechanisms of zinc-blende ZnSe NWs for three different tensile strain rates along the (**a**) [100], (**b**) [110], and (**c**) [111]-orientation.

To explain the strain rate sensitivity along different crystal orientations, we first looked at the potential energy curves for three different crystal orientations in this section. Figure S4a–c (Supporting Information) show the potential energy per atom curves for [100], [110], and [111]-directed zinc-blende ZnSe NWs as a function of strain for different strain rates. As seen in the figures, for all three different crystal orientations, the potential energy curve for different strain rates shows a similar nature before reaching the final rupturing stage. From Fig. S4b, it can be noticed that for the [110]-crystal oriented ZnSe NW with different strain rates, the fracture strain at which the potential energy curve shows a sharp decrease deviates considerably compared to the other two directions. Hence, this [110]-oriented NW demonstrates the most significant strain rate sensitivity. In addition, we have also explored the deformation mechanisms of the three different crystal-oriented ZnSe NWs for three different strain rates for a qualitative and quantitative explanation of strain rate sensitivity. Figure 12a–c depict the final stage deformation mechanisms of [100], [110], and [111]-oriented zinc-blende ZnSe NWs at strain rates of 10^8^ s^−1^, 10^9^ s^−1^, and 10^10^ s^−1^, respectively. These figures show the variation in deformation behaviors for different strain rates. For simplicity, we have just elucidated the [110]-oriented NWs deformation. Figure 12b shows two dislocations slipping at two different locations of the NW when the strain rate was 10^8^ s^−1^ for the [110] orientation. When the strain rate was increased to 10^9^ s^−1^, however, only one cleavage plane was observed in the NW, which is in stark contrast to the findings along the other two orientations shown in Fig. 12a,c. Further, a combination of different cleavage planes is noticed when the strain rate increases to its highest value of 10^10^ s^−1^. They are homogeneously nucleated using dislocation slipping and deformation twinning in several places of the NW with a cascading connection. As a result, when the strain rate changes, [110]-oriented NWs exhibit highly sensitive behavior when compared to other NWs oriented along [100] and [111] orientations.

## Conclusions

In conclusion, the tensile mechanical behavior and deformation mechanism of zinc-blende ZnSe NWs have been extensively studied at the atomistic level using MD simulations. The fracture stress, fracture strain, and elastic modulus of zinc-blende ZnSe NWs has been investigated for different crystal orientations, temperatures, and strain rates. Although zinc-blende ZnSe NW exhibits conventional brittle behavior, no temperature for the transition from brittle to ductile is identified. Square-shaped ZnSe NWs show greater value in terms of fracture strength and elastic modulus compared to hexagonal shapes at all considered diameters. We also found that the elastic modulus and ultimate tensile strength of the NWs are significantly negatively correlated with temperature. In terms of ultimate strength, elastic modulus, and fracture toughness, the [111] loading direction offers the highest values at all temperatures, whereas the [100] loading direction yields the lowest values. The NWs show the highest fracture strain under [100] loading directions. It is noticed for [100] orientation that the {111} planes are the deformation planes at smaller temperatures; conversely, when the temperature is increased to a higher value, the {100} plane is activated and contributes as the second principal cleavage plane. The elastic moduli of different crystal orientations at different strain rates show nearly constant values. Finally, it has been discovered that the [110]-direction exhibits a greater strain rate sensitivity than the other two orientations. When the strain rate increases to its highest value of 10^10^ s^−1^, a combination of different cleavage planes is noticed. They are homogeneously nucleated through dislocation slipping and deformation twinning in several places of the NW with a cascading connection. This work offers a comprehensive understanding of the mechanical properties and fracture mechanisms of zinc-blende ZnSe NWs, which are useful to effectively design future nanoelectromechanical systems.

## Supplementary Information

Supplementary Figures.

## Data Availability

The datasets used and/or analyzed during the current study available from the corresponding author on reasonable request.
