# Supplementary material for: Crystal orientation-dependent tensile mechanical behavior and deformation mechanisms of zinc-blende ZnSe nanowires
Source: Sci Rep. 2023 Mar 2;13:3532. doi: 10.1038/s41598-023-30601-3 (PMC9981763; doi:10.1038/s41598-023-30601-3)
Supplement: Supplementary file 1 — Supplementary Figures. [file 41598_2023_30601_MOESM1_ESM.docx]

**Supporting Information**

**Crystal orientation-dependent tensile mechanical behavior and deformation mechanisms of zinc-blende ZnSe nanowires**

**A. S. M. Jannatul Islam^a^, Md. Sayed Hasan^a^, Md. Sherajul Islam^a,*^, A. G. Bhuiyan^a^, Catherine Stampfl^b^**, **and Jeongwon Park^c,d^**

^a^Department of Electrical and Electronic Engineering, Khulna University of Engineering &Technology, Khulna 9203, Bangladesh

^b^School of Physics, The University of Sydney, New South Wales 2006, Australia

^c^Department of Electrical and Biomedical Engineering, University of Nevada, Reno, NV 89557, USA

^d^School of Electrical Engineering and Computer Science, University of Ottawa, Ottawa, ON K1N 6N5, Canada

***Corresponding authors E-mail:** sheraj_kuet@eee.kuet.ac.bd

**Figure S1**


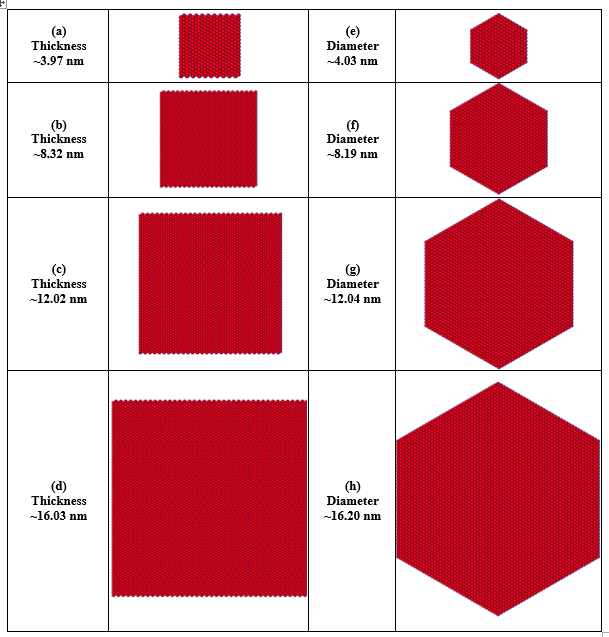


**Figure S1:** [111]-oriented square **(a, b, c, d)** and hexagonal-shaped **(e, f, g, h)** zinc-blende ZnSe NWs with diverse thickness or diameter. The considered length of all the ZnSe NWs was ~34.35 nm. For both square and hexagonal cross-sections, the [111]-oriented ZnSe NWs were surrounded by {112} and or {110} side faces.

**Figure S2**


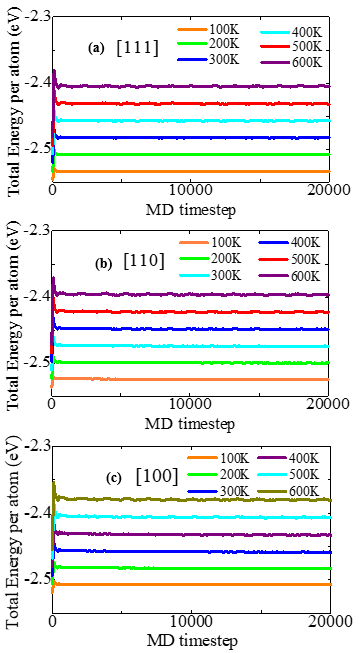


**Figure S2: Total energy per atom of (a) [111], (b) [110] and (c) [100]-oriented 15.76 nm^2^** zinc-blende **ZnSe NW for different temperatures.**

**Figure S3**


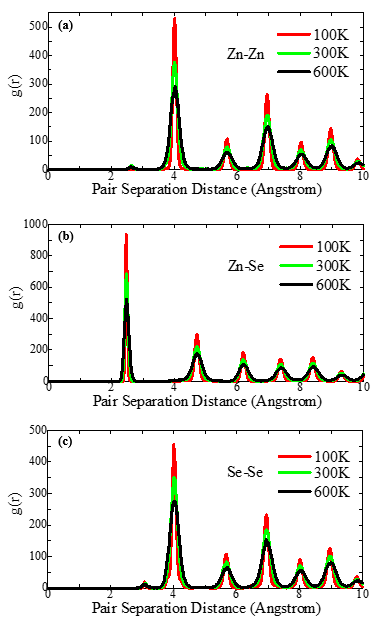


**Figure S3:** The Radial distribution function (RDF), g(r) of **(a)** Zn-Zn **(b)** Zn-Se and **(c)** Se-Se for a [100]-oriented ZnSe NW at three different temperatures.

**Figure S4**


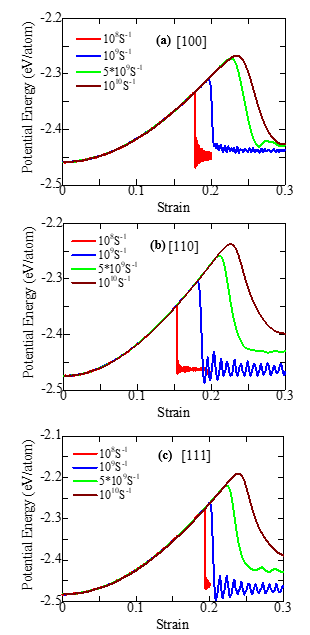


**Figure** **S4**: The potential energy per atom for **(a)** [100], **(b)** [110], and **(c)** [111]-oriented zinc-blende ZnSe NWs at 300 K.
